# EVALUATING THE IMPACT OF STAR-VA ON VETERANS’ PSYCHOTROPIC MEDICATION USE IN CLCS

**DOI:** 10.1093/geroni/igz038.2354

**Published:** 2019-11-08

**Authors:** Kevin McConeghy, Kim Curyto, Jenefer M Jedele, Jennifer Mach, Orna Intrator, Ilse R Wiechers

**Affiliations:** 1 Center of Innovation and Long-Term Services and Support, Providence VA Medical Center, Providence, Rhode Island, United States; 2 VA western NY healthcare system, batavia, New York, United States; 3 Serious Mental Illness Treatment Resource and Evaluation Center, Ann Arbor, Michigan, United States; 4 Serious Mental Illness Resource and Evaluation Center, Office of Mental Health and Suicide Prevention, Department of Veteran Affairs, Ann Arbor, New York, United States; 5 Geriatrics & Extended Care Data & Analyses Center (GEC DAC), Canandaigua VAMC, Canandaigua, New York, United States; 6 Office of mental health and suicide prevention, uS department of veteran affairs, menlo park state, California, United States

## Abstract

The impact of STAR-VA on psychotropic drug use among residents with behavioral symptoms of dementia was evaluated through a difference-in-differences framework. STAR-VA residents enrolled 2013-2017 were evaluated longitudinally pre-post intervention. The primary outcome was the number of as needed administrations with an indication of ‘anxiety’ or ‘agitation’. The analytical cohort included 214 training cases and 1,870 controls from untrained sites meeting eligibility criteria. STAR-VA cases were less white (48% vs. 54%), less black (11% vs. 14%), and had significantly longer median length of stay (830 vs. 261 days), respectively. STAR-VA cases had on average 3.5 as needed doses/month of psychotropic medication before the intervention and 1.7 after, controls averaged 1.8 doses/month. After adjustment for person-time-fixed effects, enrollment was associated with 55% (95% CI:30, 68) reduction or an average 0.8 as needed psychotropic doses/month. Findings demonstrate effectiveness in decreasing as-needed psychotropic drug use among CLC residents, supporting continued implementation of STAR-VA.

